# Mechanism of miR-30b-5p-Loaded PEG-PLGA Nanoparticles for Targeted Treatment of Heart Failure

**DOI:** 10.3389/fphar.2021.745429

**Published:** 2021-09-30

**Authors:** Yu Ren, Xiao Wang, Hongyu Liang, Wenshuai He, Xingsheng Zhao

**Affiliations:** ^1^ Scientific Research Department, Inner Mongolia People’s Hospital, Hohhot, China; ^2^ Cardiology Department, Inner Mongolia People’s Hospital, Hohhot, China

**Keywords:** PEG-PLGA, mir-30b-5p, heart failure, inflammatory related factor, TGFBR2

## Abstract

**Objective:** Exploring the effectiveness of miR-30b-5p-loaded PEG-PLGA nanoparticles (NPs) for the treatment of heart failure and the underlying mechanism.

**Methods:** PEG-PLGA characteristics with different loading amounts were first examined to determine the loading, encapsulation, and release of miR-30b-5p from NPs. The effects of miR-30b-5p NPs on cardiac function and structure were assessed by immunofluorescence, echocardiography, HE/Masson staining, and TUNEL staining. The effects of NPs on the expression of factors related to cardiac hypertrophy and inflammation were examined by RT-PCR and western blotting, and the mechanism of miR-30b-5p treatment on heart failure was explored by dual luciferase reporter assay and RT-PCR.

**Results:** The size of PEG-PLGA NPs with different loading amounts ranged from 200 to 300 nm, and the zeta potential of PEG-PLGA NPs was negative. The mean entrapment efficiency of the NPs for miR-30b-5p was high (81.8 ± 2.1%), and the release rate reached 5 days with more than 90% release. Distribution experiments showed that NPs were mainly distributed in the heart and had a protective effect on myocardial injury and cardiac function. Compared with a rat model of cardiac failure and miR-30b-5p-non-loaede NP groups, the expression of cardiac hypertrophy markers (ANP, BNPβ-MHC) and inflammatory factors (IL-1β, IL-6) were significantly decreased. Dual luciferase reporter assay assays indicated that miR-30b-5p exerted its effects mainly by targeting TGFBR2.

**Conclusion:** PEG-PLGA NPs loaded with miR-30b-5p improved cardiac function, attenuated myocardial injury, and regulated the expression of factors associated with cardiac hypertrophy and inflammation by targeting TGFBR2.

## Introduction

Heart failure (HF) is a complex clinical syndrome caused by initial myocardial damage for a variety of reasons, resulting in changes in the structure and function of the heart, leading to a reduced pumping function of (Parada et al., 2012). The main clinical manifestations were hemodynamic changes, such as congestion of the pulmonary and/or systemic circulation and insufficient tissue blood perfusion. The incidence rate of heart failure is rising, which is closely related to the aging of the population (Seferovi et al., 2021). At present, most of the drugs for heart failure lack tissue specificity and are less absorbed by cardiac cells, which leads to poor efficacy (Dharmarajan and Rich, 2017). Therefore, it is of great clinical significance to construct a novel drug-loading system that can increase the amount of drug uptake by cardiomyocytes.

With the development of nanotechnology, nanomaterials have gained extensive attention in drug loading, diagnosis, medical imaging, and other fields. Poly (lactic-co-glycolic acid) (PLGA), formed after co-condensation of lactic and glycolic acids, has good biocompatibility and biodegradation properties, and its degradation rate can be adjusted by changing the ratio of comonomers. Its application in the pharmaceutical field has been approved by the US Food and Drug Administration (FDA) and the European Medicines Agency (EMA), and is currently the most attractive polymer carrier (Pedram and Azita, 2017; Xu et al., 2017).

Polyethylene glycol (PEG) is a water-soluble polyether obtained by the gradual addition of water, ethylene glycol, and ethylene oxide. It has good biocompatibility and immunogenicity. In recent years, PEG has been introduced into polymer carriers as a hydrophilic component. The most commonly used method is to form a PEG-PLGA block copolymer using a block or graft, which can effectively improve drug solubility, increase drug stability, and reduce immunogenicity (Park et al., 2009).

The application of the polymer PEG-PLGA in cancer, cardiovascular and cerebrovascular diseases, and immunological diseases as well as its application as a carrier in preparations has been reported (Wang et al., 2011; Saneja et al., 2019). However, PEG-PLGA as an miRNA carrier for the treatment of heart failure has not been studied. In this study, the loading and release of miR-30b-5p by PEG-PLGA nanoparticles (NPs) were investigated. The distribution of NPs *in vivo* and its therapeutic effect on heart failure were investigated using animal experiments. Furthermore, the molecular mechanism of miR-30b-5p loaded onto NPs in heart failure was further explored.

## Material and Methods

### PLGA-PEG-COOH Capsule Preparation

Non-loaded, single-loaded indole-green (ICG), co-loaded miR-30b-5p and ICG PLGA-PEG-COOH capsules were prepared by water and oil emulsification. First, 150 mg PLGA polymer was dissolved in 1.5 ml dichloromethane, and 18 g N-hydroxysuccinimide (NHS) and diethyl carbonate (DEC) were added to the solution. After reaction for 4 h at room temperature, the reaction mixture was poured into cold methanol/Et2O (1:1), centrifuged at 12,000 rpm for 1 min, and the precipitate was vacuum dried to obtain the PLGA-NHS ester. The dried PLGA-NHS ester was dissolved in chloroform and mixed with NH2-PEG-COOH (MW 3400) and diisopropylethylamine. The reaction was continued at room temperature for 24 h. The reaction mixture was poured into cold MEOH/Et2O (1:1), washed twice, and vacuum dried to obtain PLGA-B-PEG-COOH. Ten milligrams of PLGA-B-PEG copolymer and emulsifier 80 were dissolved in 1 ml of dichloromethane. Anti-miR-30b-5p bound to Cy5 was added into 0.3 ml DNase/RNase free water and mixed with spermidine at a ratio of 15:1. ICG (0.5 mg) was dissolved in 0.1 ml DMSO, and the mixture was added to the above organic solution and mixed until the solution became uniform and clear. The arginine antisense miR-30b-5p complex was added to the organic solution above, and then ultrasonically treated for 60 s in an ice bath at 40% amplitude to form the first emulsion. Then, the first emulsion drops were added to 5 ml of 1% PVA (W/V) autoclaved double-distilled water. After a slight stirring, it was ultrasonically processed for 60 s in an ice bath at 40% amplitude to form the two-level emulsion. The mixture was stirred at room temperature for 3 h to vaporise the organic solvent and enhance the NPs, and the enhanced NPs were filtered, disinfected, and washed.

### Cardiac Homing Peptide Coupling

With the development of phage display technology, hundreds of homing peptides and their derivatives have been developed. The cardiac homing peptide, which is a polypeptide that can specifically target the heart muscle, was identified by phage screening technology, and it can be used as a drug delivery vehicle targeting heart muscle disease (Adam V et al., 2018). One mL of PLGA-PEG-COOH capsules loaded with ICG and miR-30b-5p (10 mg/ml) were centrifuged, dispersed into 0.5 ml MES (100 mM, pH 4.8), and cardiac homing peptide (CHP) heart homing peptide (1,000 μg) was added for adsorption at 37°C for 1 h. Eighty μl EDC solution were added and the solution incubated overnight at 37°C. The concentration of CHP in the supernatant was measured and the coupling amount was calculated.

### Particle Size and Zeta Potential Detection of NPs

NPs were divided into no-loaded PLGA-PEG-COOH NPs, PLGA-PEG-COOH NPs loaded with ICG, PLGA-PEG-COOH NPs co-loaded with ICG and miR-30b-5p, ICG-loaded as well as ICG and miR-30b-5p co-loaded PLGA-PEG-COOH NPs were conjugated with CHP. The size of NPs was measured using a nanoparticle size analyser (Brookhaven, United States), and the zeta potential was measured using a zeta potential meter (Brookhaven, United States).

### The Structure of NPs Was Observed by Transmission Electron Microscope

0.5 mg NPs were added to 4 ml of ddH_2_O and mixed well, and one drop was then added to a copper wire with a sterile pipette, and allowed to dry at room temperature. The same volume of phosphotungstic acid was stained and observed under a transmission electron microscope.

### Determination of Drug Loading, Entrapment Efficiency of NPs

MiR-30b-5p was mixed into 100, 50, 25, 12.5, 6.25, 3.125, 1.562, 0.781, 0.39, and 0.1 μmol/L solutions. The absorbance values at 260 and 280 nm were measured using a spectrophotometer, and standard curves were established. NPs loaded or not loaded with miR-30b-5p were prepared to measure the amount of free RNA in the supernatant after centrifugation, and the encapsulation rate was calculated as the difference between miR-30b-5p and total RNA. RNA encapsulation rate = (total RNA amount - free RNA amount)/total RNA amount ×100%. Drug loading of RNA = (total RNA amount-free RNA amount)/total nanoparticle mass ×100%.

### The Release Rate of miR-30b-5p From NPs Was Detected by Dialysis

PLGA-PEG-COOH NPs loaded with ICG, miR-30b-5p, and CHP (40 mg/ml, 1 ml) were weighed and placed into a dialysis bag (8 K-14K), which was placed into a centrifuge tube containing 5 ml of release medium (0.02 mol/L PBS containing 1% SDS [pH 7.4] ) and vibrated using a shaker at 37°C and 100 rpm. At different time points of 0 h (control), 0.5, 1, 2, 4, 19, 24, 48, 72, 96, and 120 h, 1 ml of the release solution was taken, and the absorbance was measured using a UV spectrophotometer after filtration through a 0.45 μm membrane.

### Cell Culture

Human cardiac myocytes (HCM) were obtained from the American Type Culture Collection (ATCC) and cultured in low-glucose DMEM containing 10% foetal bovine serum (FBS). The concentration of penicillin was 100 U/mL, the concentration of streptomycin was 100 U/mL, and the cells were cultured in an incubator at 37°C and 5% CO_2_. At 80% confluence, the cells were digested 0.25% trypsin and subcultured.

### CCK-8 Method Was Used to Detect the Effect of NPs on the Proliferation of Cardiomyocytes

Blank, miR-30b-5p-loaded or miR-30b-5p-non-loaded NPs were used to treat cardiomyocytes for 24 h. Cells in each group were seeded in 96 well plates and incubated for 24, 48, and 72 h at 37°C. Then, 100 μl of medium was added to 10 μl CCK-8 (Beyotime, Shanghai, China) and incubated at 37°C for 4 h. The absorbance (OD value) was measured with a microplate reader at a wavelength of 450 nm, and the process was repeated five times. The average value was used to calculate cell viability.

### Acute Myocardial Infarction Rat Model Construction

Left coronary artery permanent ligation was used to establish a heart failure model in rats. Adult male Wistar rats were anaesthetized with sodium pentobarbital and fixed. The median incision was about 0.8 cm upward from 0.4 cm above the sternum. The trachea was separated layer-by-layer until exposure. After tracheal intubation, a small animal ventilator was used. The tidal volume was 30 ml/kg and the mechanical ventilation frequency was 30–40/min. Approximately 3–4 cm of skin was cut longitudinally (0.4 cm from the right midline at the left sternal border, and the subcutaneous tissue, muscle layer, and rib and intercostal muscles were separated layer by layer. The apical beating of rats was observed the 4th intercostal muscle was bluntly separated along the 4th intercostal space, and the heart was exposed by hemostat distraction. The heart was extruded out of the thoracic cavity, and a 6/0 suture was inserted through the myocardium 4 mm below the root of the left atrial ear, and a needle was inserted in the direction of the interventricular septum to complete the ligation of the left anterior descending branch. The heart was retracted into the thorax, and an electrocardiogram was observed with the ST segment arched upward as a marker of successful ligation. In the sham group, the needle was passed through the myocardium with a 6/0 suture at the same site toward the interventricular septum, but was not ligated, and the electrocardiogram was observed without ST segment arched upward elevation changes. This animal experiment was approved by the ethics committee of our hospital (No.2020018).

### Immunofluorescence Detection of NP Distribution and Delivery *In Vivo*

The heart failure model rats were anaesthetized by intraperitoneal injection of 1% sodium pentobarbital (40 mg/kg), and 1 ml Dil-labeled NPs were injected through the tail vein. Forty-eight hours after injection, the heart, the liver, the spleen, the lung, and the kidney were harvested and frozen. After fixing in 4% paraformaldehyde for 15 min, the NPs were stained with DAPI dye (Sigma, United States) for 10 min and washed with PBS three times. The tissue distribution of the NPs was observed using a confocal laser scanning microscope.

### Cardiac Function Testing in Model Rats

After the success of model construction, the rats were anaesthetized using sodium pentobarbital *via* the ear edge vein, and the preputial skin was disinfected. A left anterolateral incision was made, the 3rd and 4th ribs were cut, the pericardium was incised, and the circumflex artery of the left coronary artery toward the dorsal side was exposed. 0.8 ml of the gene nanoparticle suspension were injected intramyocardially at multiple points under direct vision with a syringe at the distal end of the rotating bifurcation. The pericardium, ribs, muscles, and incision were sutured layer by layer. Left ventricular posterior wall thickness at end diastolic (LVPWD), end-systolic left ventricular posterior wall thickness (LVPWS), interventricular septum end diastolic thickness (IVSD), interventricular septum end systolic thickness (IVSS), left ventricular ejection fraction (LVEF), and left ventricular short axis shortening rate (LVFS) were recorded by electrocardiography to observe the cardiac function of rats in each group.

### H and E Staining of Myocardial Tissue

After anaesthesia, hearts tissues were fixed in neutral formaldehyde for 24 h. The heart tissues were embedded in paraffin and sectioned (4–5 μm) for HE staining. The conventional paraffin sections were soaked in xylene for 30 min, dehydrated with 100, 95, 85, and 75% ethanol solutions for 5 min, followed by water washing, hematoxylin staining for 10 min, soaking in 1% hydrochloric acid, and rinsing with distilled water. Eosin staining (Dakwei, Beijing, China) for 3 min, 75, 85, 95, and 100% ethanol solution for 2 min, xylene transparent treatment, drop neutral resin cover glass, and placed under a microscope to observe the results.

### Masson Staining

Masson staining was used to observe collagen deposition in the myocardial tissue. Paraffin sections of the myocardial tissue were dewaxed and hydrated. Ponceau acid fuchsin solution was used for staining for 5–10 min. After washing with distilled water, 1% phosphomolybdic acid solution was used for staining for 5 min. After removing the dye solution, the sections were stained with aniline blue for 5 min, washed with distilled water, and treated with 1% glacial acetic acid for 1 min. The samples were dehydrated with 95% ethanol and anhydrous ethanol, transparent with xylene, and sealed. Five visual fields were randomly selected from each specimen and photographed.

### Detection of Cardiomyocyte Apoptosis by the TUNEL Method

Three sections of each specimen were randomly selected and operated according to the instructions of the TUNEL Kit (Roche, Switzerland). After DAB staining, sections were observed under a 400× light microscope. Apoptotic cells were identified when brown-yellow granules appeared in the nucleus. Five non-overlapping randomly selected fields were photographed, the images were analyzed, and the apoptotic index was calculated.

### Real Time Fluorescent Quantitative Polymerase Chain Reaction

Total RNA was extracted with TRIzol reagent (Invitrogen, MA, United States), and the concentration and purity of total RNA were measured by spectrophotometry and agarose gel electrophoresis. Total RNA (1 μg) was reverse transcribed *in vitro* to generate cDNA (Takara, Japan). The reaction conditions were 42°C for 60 min and 70°C for 5 min, and the cDNA products were stored at −20°C. SYBR Green Master Mix (Thermo Fisher Scientific, United States) was used to determine gene expression levels. The reaction conditions were set as follows: pre-denaturation at 95°C for 5 min, denaturation at 95°C for 30 s, annealing at 60°C for 30 s, and extension at 72°C for 30 s, for 40 cycles. Relative gene quantification was performed using GAPDH and U6 as internal reference genes and the 2^−ΔΔCT^ method on CFX Manager 3.0. The RT-PCR primer sequences are listed in Table 1.

**TABLE 1:** RT-PCR primer sequences.

Gene names	Primer sequences (5′-3′)
ANP Forward primer	AGG​AGA​AGA​TGC​CGG​TAG​AAG
ANP Reverse primer	AGA​GCC​CTC​AGT​TTG​CTT​TTC
BNP Forward primer	GCTCTCAAAGGACCAAGG
BNP Reverse primer	AAACAACCTCAGCCCGTC
β-MHC Forward primer	CAC​TCC​AGA​AGA​GAA​GAA​CTC​CA
β-MHC Reverse primer	ATA​CTC​GTT​GCC​CAC​TTT​GAC​T
IL-1β Forward primer	ATA​GCA​GCT​TTC​GAC​AGT​GAG
IL-1β Reverse primer	GTC​AAC​TAT​GTC​CCG​ACC​ATT
IL-6 Forward primer	TTCCCTACTTCACAAGTC
IL-6 Reverse primer	CTAGGTTTGCCGAGTAGA
miR-30b-5p Forward primer	GCG​CTG​TAA​ACA​TCC​TAC​AC
miR-30b-5p Reverse primer	GTGCAGGGTCCGAGGT
TGFBR2 Forward primer	GGG​ATT​GCC​ATA​GCT​GTC​AT
TGFBR2 Reverse primer	TTG​TCG​CTG​AAA​TCC​ATG​AG
U6 Forward primer	CTC​GCT​TCG​GCA​GCA​CAT​ATA​CT
U6 Reverse primer	ACG​CTT​CAG​AAT​TTG​CGT​GTC
GAPDH Forward primer	TGT​GTC​CGT​CGT​GGA​TCT​GA
GAPDH Reverse primer	TTG​CTG​TTG​AAG​TCG​CAG​GAG

### Western Blot

Total proteins from each group were extracted with RIPA lysis buffer (Biyuntian, Beijing, China). A BCA protein assay kit (Thermo, United States) was used to determine the total protein concentration. After SDS-PAGE, the proteins were transferred to a PVDF membrane (Millipore, United States) and blocked with 5% skimmed dry milk solution at room temperature for 2 h. Then, the prepared primary antibodies against Col-I, TGFBR2, and GAPDH (rabbit anti-monoclonal antibody, 1:1,000) were added and incubated overnight at 4°C. The membrane was washed three times with TBST for 5 min each time. The corresponding secondary antibody (goat versus rabbit, 1:2000) was added and incubated for 1–1.5 h, and then the membrane was washed with TBST–3–4 times. An ECL chromophore (Aillipore, MA, United States) was used to visualize protein bands, and the results were analysed using Quantum One software, in which GAPDH was used as an internal reference.

### Double Luciferase Activity Determination

The luciferase activity assay verified the binding of miR-30b-5p to TGFBR2. The binding sites predicted by the software were inserted into the PMIR-Report luciferase REPORT vector, which were named WT-TGFBR2 and MUT-TGFBR2, respectively. Then the luciferase gene assay was used to co-transfect the cells with a 200 ng luciferase reporter gene vector and 25 ng PRL-TK (expressing luciferase as an internal control) using Lipofectamine 2000. Twenty-four h after luciferase transfection, the luciferase reporter gene assay system (Promega) was used to analyse enzyme activity.

### Statistical Analysis

SPSS 23.0 and Origin 9.1 statistical softwares were used for data analysis. The measurement data are expressed as the mean ± standard deviation (‾ x ± s). One-way ANOVA was used for comparison among groups, and the SNK-Q test was used for pairwise comparisons between groups. Statistical significance was set at *p* < 0.05.

## Results

### Preparation and Analysis of PLGA-PEG-COOH Capsules

#### Dynamic Light Scattering (DLS) Results

**FIGURE 1:**
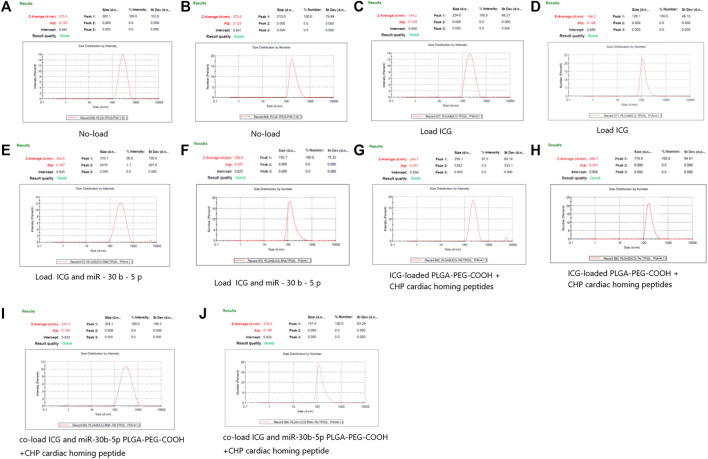
DLS results of different PLGA-PEG-COOH nanoparticles **(A,B)** DLS results of no - loaded PLGA-PEG-COOH nanoparticles. A and B are the results of Intensity and Number respectively. **(C,D)** DLS results of PLGA-PEG-COOH nanoparticles loaded with ICG. C and D are the results of Intensity and Number respectively. **(E,F)** DLS results of PLGA-PEG-COOH nanoparticles loaded with ICG and miR-30b-5p. E and F are the results of Intensity and Number respectively. **(G,H)** DLS results of ICG-loaded PLGA-PEG-COOH nanoparticles coupled with CHP. G and H are the results of Intensity and Number respectively. **(I,J)** DLS results of the conjugation of CHP by PLGA-PEG-COOH nanoparticles co-loaded with ICG and miR-30b-5p. I and J are the results of Intensity and Number respectively.

The average particle size of empty non-loaded PLGA-PEG-COOH NPs, ICG-loaded PLGA-PEG-COOH NPs, and that of co-loaded ICG and miR-30b-5p PLGA-PEG-COOH NPs was 273.5 ± 23.3, 194.2 ± 20.1, and 280.8 ± 31.1 nm, respectively. The mean particle sizes of CHP-conjugated PLGA-PEG-COOH NPs loaded with ICG, or co-loaded with ICG and miR-30b-5p were 248.7 ± 25.5 and 278.3 ± 26.4 nm, respectively (Figure 1).

#### Zeta Potential Results

**FIGURE 2:**
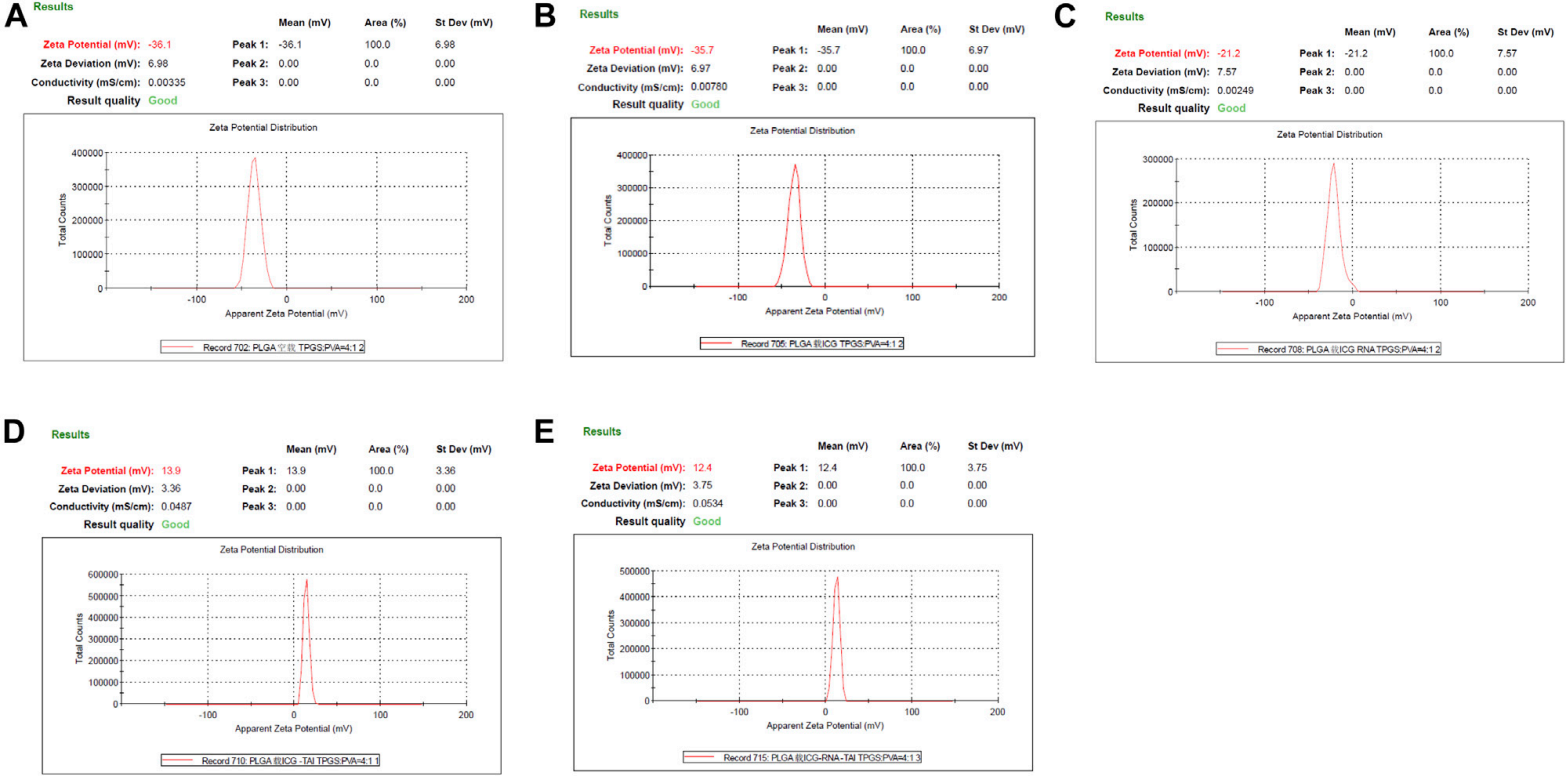
Zeta potential of PLGA-PEG-COOH nanoparticles in different groups **(A)** Zeta potential results of no-load PLGA-PEG-COOH nanoparticles. **(B)** Zeta potential results of ICG-loaded PLGA-PEG-COOH nanoparticles. **(C)** Zeta potential results of PLGA-PEG-COOH nanoparticles co-loaded with ICG and miR-30b-5p. **(D)** Zeta potential results of ICG-loaded PLGA-PEG-COOH nanoparticle coupling CHP. **(E)** Zeta potential results of the coupling of CHP with PLGA-PEG-COOH nanoparticles loaded with ICG and miR-30b-5p.

The zeta potential of the non-loaded PLGA-PEG-COOH NPs, PLGA-PEG-COOH NPs loaded with ICG, and PLGA-PEG-COOH NPs loaded with ICG and miR-30b-5p was − −36.1 ± 2.1, −35.7 ± 1.9, and −21.2 ± 1.2 mV, respectively. The zeta potential of CHP-cojugated PLGA-PEG-COOH NPs loaded with ICG an PLGA-PEG-COOH NPs loaded with ICG and miR-30b-5p was 13.9 ± 0.6 and 12.4 ± 0.7 mV, respectively (Figure 2).

#### Results of Transmission Electron Microscope

**FIGURE 3:**
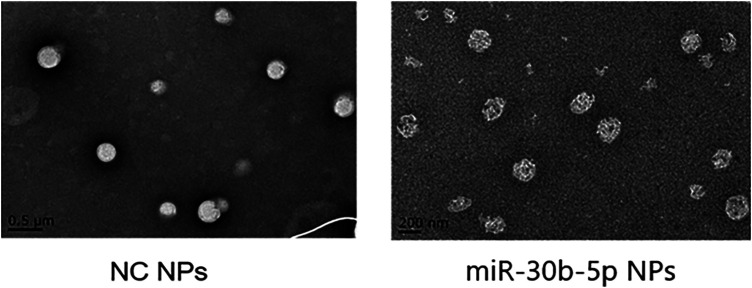
Observation of NPs by transmission electron microscopy. In transmission electron microscopy (TEM), NPs loaded with miR-30b-5p appear as a higher electron density core surrounded by a lower density shell.

Transmission electron microscopy was performed on mir-30b-5p-loaded and mir-30b-5p-non-loaded NPs, and the results are shown in Figure 3.

#### Drug Loading, Encapsulation Rate and Release Results of NPs

**FIGURE 4:**
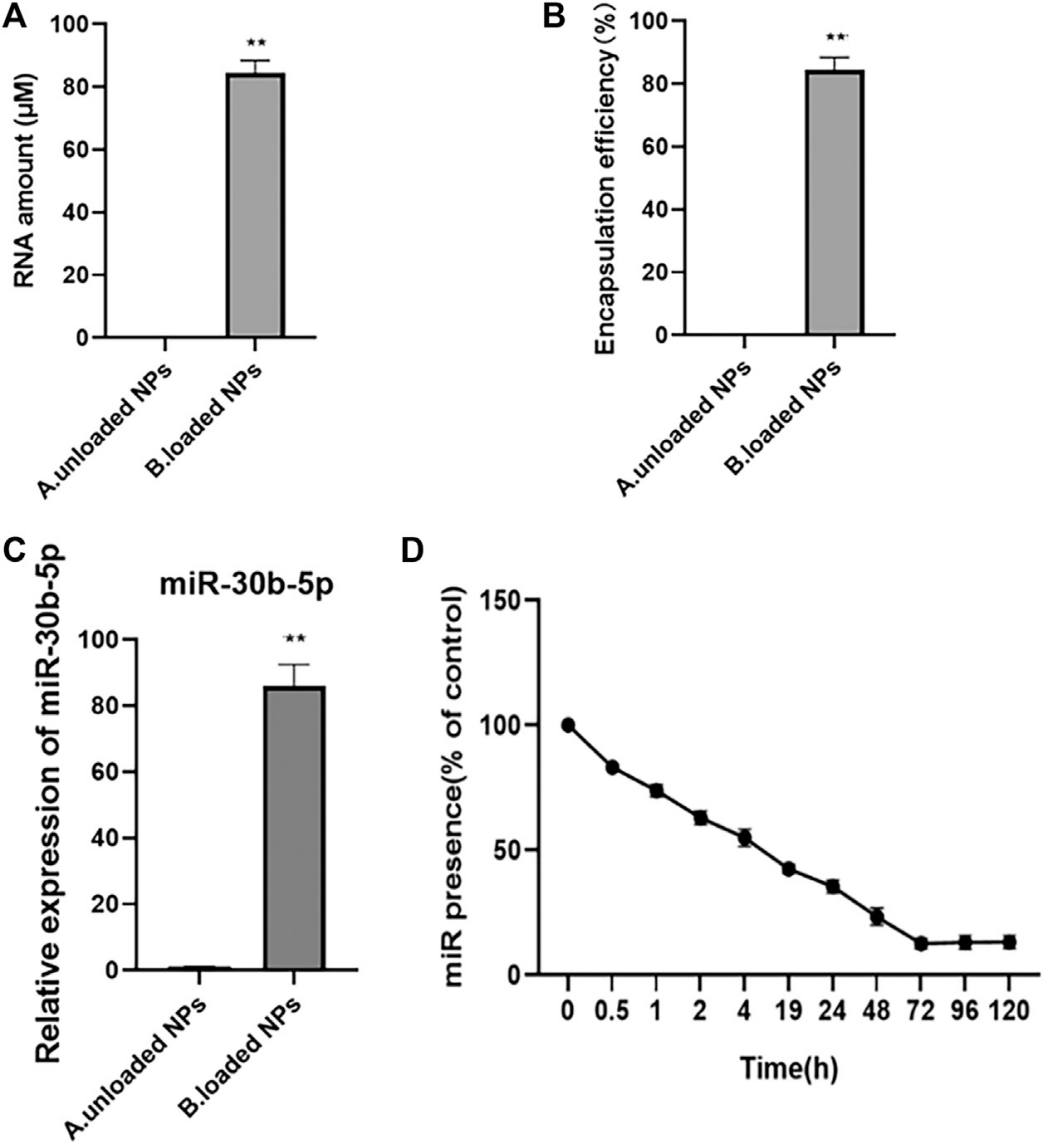
The loading, encapsulation rate and release of miR-30b-5p by nanoparticles **(A)**. Quantitative results of nanoparticles loaded or not with miR-30b-5p. **(B)**. Results of encapsulation efficiency of nanoparticles loaded or not with miR-30b-5p. **(C)**. Expression difference of miR-30b-5p in nanoparticles loaded with or without miR-30b-5p. **(D)**. Release of miR-30b-5p from nanoparticles within 120 h *p < 0.05, **p < 0.01.

The loading, encapsulation rate, and release of miR-30b-5p by NPs were determined, and the results are shown in Figure 4. The results showed that the level of NPs RNA loaded with miR-30b-5p was significantly higher than that without miR-30b-5p (*p* < 0.05). The encapsulation rate of NPs loaded with miR-30b-5p was significantly higher than that of NPs non-loaded with miR-30b-5p (*p* < 0.05), and the mean entrapment efficiency of the NPs for miR-30b-5p was 81.8 ± 2.1%. The levels of miR-30b-5p in NPs loaded with miR-30b-5p was significantly higher than that in non-loaded NPs (*p* < 0.05). In addition, within 120 h, the release of miR-30b-5p decreased gradually with time. The release of miR-30b-5p from NPs was rapid in the initial 24 h and levelled off after 48 h. The sustained release was as long as 5 days, and the cumulative release was more than 90%.

### Safety of NPs

#### Effect of NPs on Cardiomyocyte Proliferation

**FIGURE 5:**
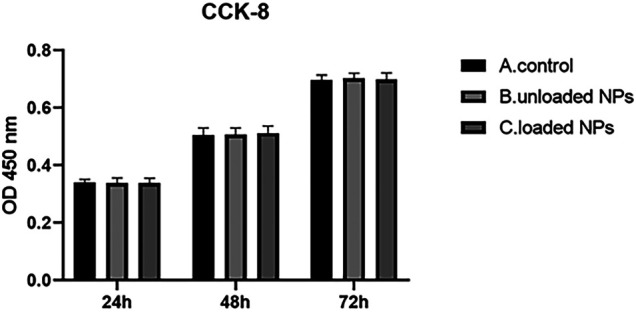
CCK-8 assay for proliferation effect of nanoparticles on cardiomyocytes.

The proliferation effect of NPs on cardiomyocytes was detected by the CCK-8 assay (Figure 5), and the results showed that there was no obvious difference between miR-30b-5p-loaded NPs and non-loaded NPs, blank group (*p* > 0.05), which indicated that miR-30b-5p-loaded NPs had no effect on cell proliferation.

#### Distribution and Delivery of NPs *In Vivo*

**FIGURE 6:**
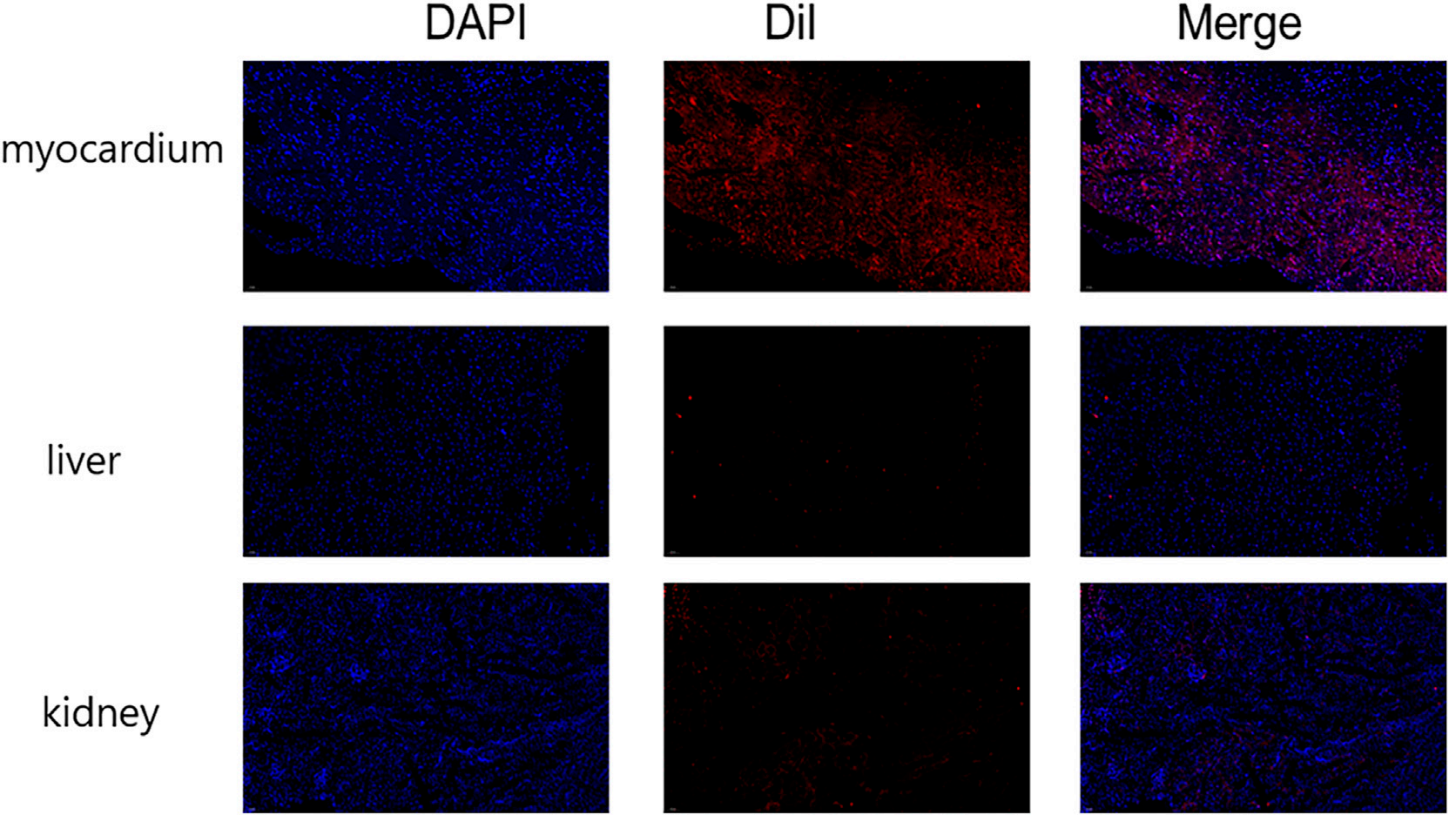
Immunofluorescence detection of nanoparticle distribution *in vivo*. The fluorescence intensity was greatest in the heart and less intense in the liver and kidney. (n = 10).

Immunofluorescence was used to detect the distribution of NPs *in vivo*. The results showed that the fluorescence intensity of NPs was the highest in the heart, and the fluorescence signals of the liver and the kidney were lower, indicating that NPs were mainly distributed in the heart (Figure 6).

### Therapeutic Effect of NPs on Heart Failure

#### Echocardiographic Findings

**FIGURE 7:**
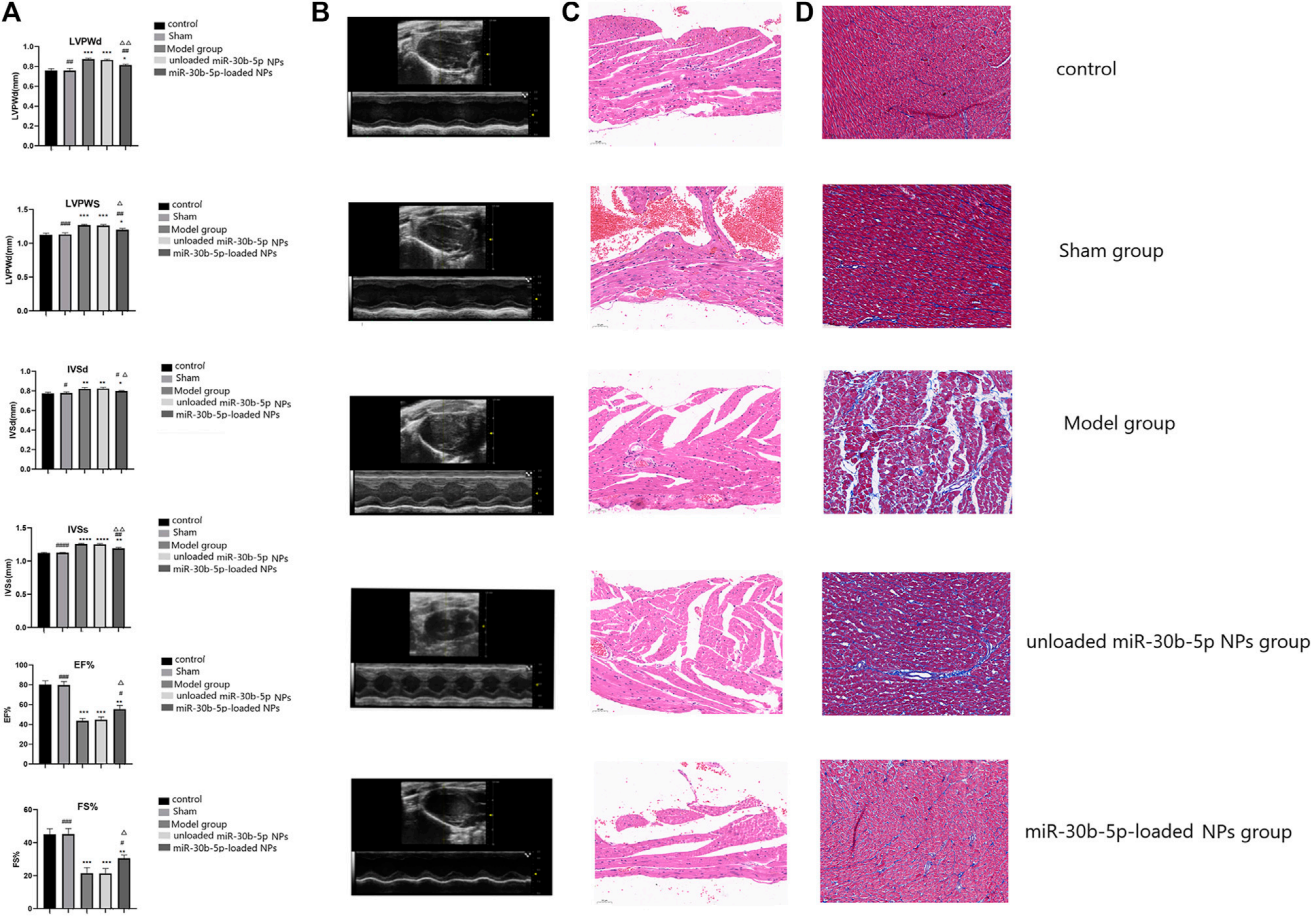
Effect of miR-30b-5p-loaded nanoparticles on cardiac function and myocardial injury. **(A,B)**. Effects of nanoparticles on cardiac function. There were 10 rats in each group. Compared with the control group, *p < 0.05, **p < 0.01; Compared with the model group, ^#^ *p* < 0.05, ^##^ *p* < 0.01; Compared with unloaded miR-30b-5p NPs group, ^△^ *p* < 0.05, ^△△^ *p* < 0.01. **(C)**. HE staining results of different loaded nanoparticles in myocardial injury. **(D)**. Masson staining results of different loaded nanoparticles in myocardial injury. (n = 10).

In these experiments the animals were grouped as follows control, sham, model, miR-30b-5p-non-loaded NP, and miR-30b-5p-loaded NP groups. Cardiac function was assessed by ECG measurement of LVPWD, LVPWS, IVSD, IVSS, EF% and FS% (Figures 7A,B). Compared with the control group, LVPWD, LVPWS, IVSD and IVSS in the model group, miR-30b-5p-non-loaded or miR-30b-5p-loaded NP groups were significantly higher than those in the control group (*p* < 0.05). In addition, LVPWD, LVPWS, IVSD, and IVSS of the miR-30b-5p-loaded NP group were significantly lower than those of the model group and the miR-30b-5p-non-loaded NP group (*p* < 0.05). Compared with the control group, EF% and FS% in the model and the miR-30b-5p-non-loaded or miR-30b-5p-loaded NP groups were significantly lower than those in the control group, and EF% and FS% in the miR-30b-5p-loaded NP group were significantly lower than those in the model and miR-30b-5p-non-loaded NP group (*p* < 0.05). The results indicated that the heart function of rats in the heart failure model group treated with miR-30b-5p-loaded NPs was significantly better than that of rats in the heart failure model group treated withmiR-30b-5p-non-loaded NPs.

#### Results of HE Staining

According to the morphology of myocardial cells results, the myocardial fibres of rats in the control and the sham operation groups were arranged neatly, and the nuclei and cytoplasm were uniformly stained. The myocardial cells in the model and the miR-30b-5p-non-loaded groups were significantly sparse and hypertrophic, and the myocardial fibres became coarser and more spaced, and some of them were broken and arranged disorderly. The degree of myocardial injury in the miR-30b-5p-loaded group was less than that in the model and the miR-30b-5p-non-loaded groups. The heart failure model group rats that had been treated with miR-30b-5p-loaded NPs had significantly less cardiac damage than the group treated with miR-30b-5p-non-loaded NPs (Figure 7C).

#### Masson Staining Results

Severe ventricular fibrosis in the model and miR-30b-5p-non-loaded NPs groups with hypertrophied cardiomyocytes with indistinct margins and increased blue colour in the interstitial space indicated hyperplasia of collagen fibres. The miR-30b-5p-loaded NP group had far fewer fibres than the model and the miR-30b-5p-non-loaded groups. Occasionally, blue-stained collagen fibrils were observed between cardiac fibres in the control and sham-operated hearts. The heart injury of rats in the heart failure model group treated with miR-30b-5p-loaded NPs was significantly lower than that of rats in the heart failure model group treated with miR-30b-5p non-loaded NPs (Figure 7D).

#### Cardiomyocyte Apoptosis

**FIGURE 8:**
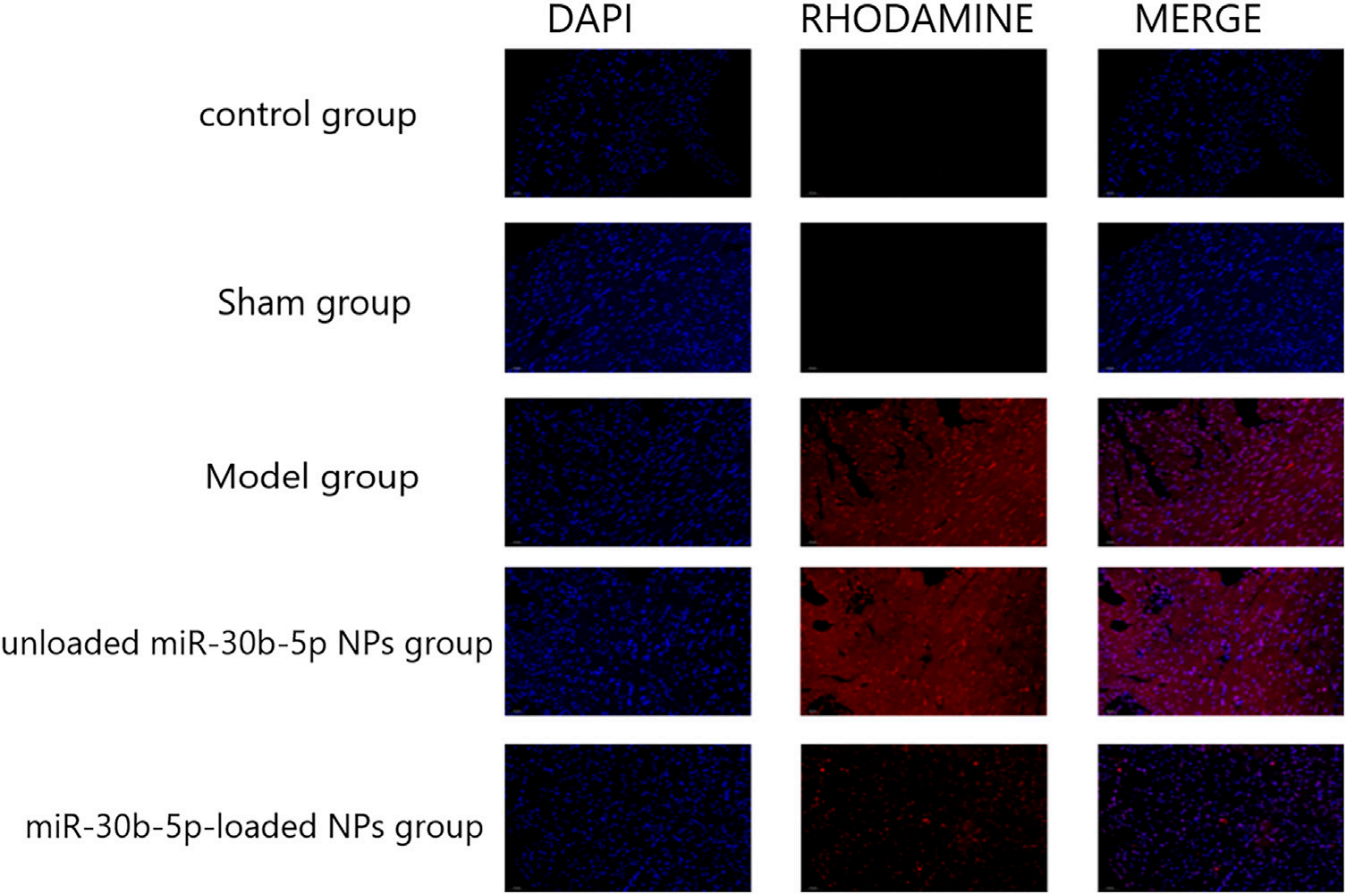
TUNEL staining was used to detect the effect of different loaded nanoparticles on cardiomyocyte apoptosis. (n = 10).

The fluorescence signal was the strongest in the model and miR-30b-5p-non-loaded groups, followed by the miR-30b-5p-loaded group, while the control and sham groups showed the weakest fluorescence signal. The apoptosis rate of cardiomyocytes in the heart failure model group rats treated with miR-30b-5p-loaded NPs was lower than that in heart failure rats treated with miR-30b-5p-non-loaded (Figure 8).

### Effect of NPs on Cardiac Hypertrophy and Inflammation

**FIGURE 9:**
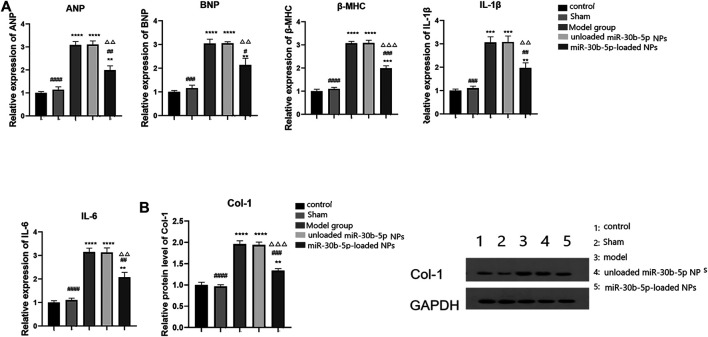
Effects of different loaded nanoparticles on the expression of markers of cardiac hypertrophy and inflammatory factors. **(A)**. Detection of myocardial hypertrophy markers and inflammatory factors by RT-PCR. Compared with the control group, *p < 0.05, **p < 0.01; Compared with the model group, ^#^ *p* < 0.05, ^##^ *p* < 0.01; Compared with unloaded miR-30b-5p NPs group, ^△^ *p* < 0.05, ^△△^ *p* < 0.01. **(B)**. The expression of Col-1 protein was detected by Western blot. Compared with the control group, *p < 0.05, **p < 0.01; Compared with the model group, ^#^ *p* < 0.05, ^##^ *p* < 0.01; Compared with unloaded miR-30b-5p NPs group, ^△^ *p* < 0.05, ^△△^ *p* < 0.01.

RT-PCR was used to detect the expression of myocardial and inflammatory markers. The results are shown in Figure 9A. The expression levels of cardiac hypertrophy markers (ANP, BNP, and β-MHC) and inflammatory factors (IL-1β and IL-6) increased in the model, the mir-30b-5p-non-loaded, and the mir-30b-5p-loaded groups (*p* < 0.05). However, compared with the model and mir-30b-5p-non-loaded NPs, the expression of cardiac hypertrophy markers (ANP, BNP, and β-MHC) and inflammatory factors (IL-1β, IL-6) were significantly decreased (*p* < 0.05). The protein expression of Col-1 was detected by western blotting, and the results are shown in Figure 9B. Compared with the control group, Col-1 protein expression was significantly higher in the model, the miR-30b-5p-non-loaded NP, and the miR-30b-5p-loaded NP groups (*p* < 0.05). However, Col-1 protein was significantly decreased in the miR-30b-5p-loaded NP group compared with the model group and the miR-30b-5p-non-loaded NP group.

### Molecular Mechanism of miR-30b-5p-Loaded in NPs in Heart Failure

**FIGURE 10:**
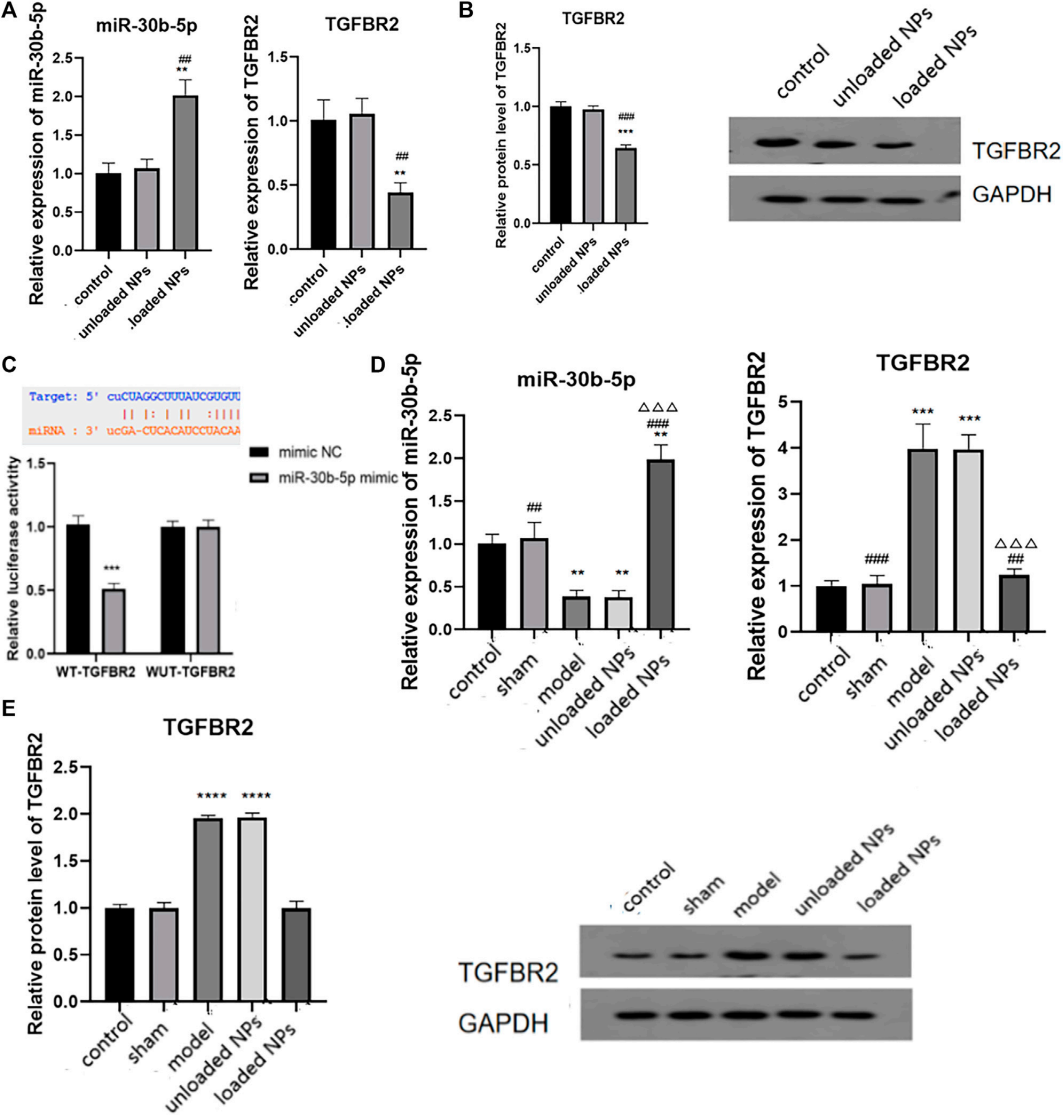
Molecular mechanism of miR-30b-5p in the treatment of heart failure. **(A,B)**. Expression difference of miR-30b-5p and TGFBR2 in different groups. **(C)**. The binding of miR-30b-5p to tgfbr2 was detected by dual luciferase activity assay. **(D,E)** Interaction between miR-30b-5p and TGFBR2 *in vivo* results. Compared with the control group, *p < 0.05, **p < 0.01; Compared with the model group, ^#^ *p* < 0.05, ^##^ *p* < 0.01; Compared with unloaded miR-30b-5p NPs group, ^△^ *p* < 0.05, ^△△^ *p* < 0.01.

Transforming growth factor β II receptor (TGFBR2) is an important component of TGF/Smad signalling. TGF must bind to the specific receptor TGFBR2 to activate downstream signal transduction pathways and play a role in myocardial injury. We identified binding sites of miR-30b-5p and TGFBR2 by bioinformatics analysis, so TGFBR2 can be a target gene of miR-30b-5p. The expression levels of miR-30b-5p and TGFBR2 in cardiomyocytes were examined by RT-PCR, and the expression of miR-30b-5p in cardiomyocytes treated with miR-30b-5p-loaded NPs was higher than that in the control group and the miR-30b-5p-non-loaded NP group (*p* < 0.05) (Figure 10A). The expression of TGFBR2 in NPs-treated cardiomyocytes with the opposite load of miR-30b-5p was lower than that in the other two groups (*p* < 0.05) (Figures 10A,B). In view of this, the correlation between the two was further explored using the luciferase activity assay, as shown in Figure 10C. Luciferase activity was significantly decreased after co-transfection of wild-type TGFBR2 and miR-30b-5p mimic, indicating the presence of binding sites. The expression of miR-30b-5p and TGFBR2 was detected in animals *in vivo*, and the results showed that the expression of miR-30b-5p in the model group and the miR-30b-5p-non-loaded group was significantly decreased compared to that in the control group. The expression of miR-30b-5p in the miR-30b-5p-loaded NPs group was significantly increased (*p* < 0.05) (Figure 10D). In contrast, compared with the control group, the mRNA and protein expression of TGFBR2 was significantly increased in the model group and the miR-30b-5p-non-loaded NP group. Compared with these two groups, mRNA and protein expression of TGFBR2 was significantly decreased in the miR-30b-5p-loaded NP group (*p* < 0.05) (Figure 10D,E).

## Discussion

As a drug carrier, PLGA greatly promotes the development of drug preparations, but its hydrophobicity allows easy clearance by mononuclear macrophages in the human body and its absorbance by the liver and spleen. However, PEG has good solubility in water and many organic solvents, and its connection with hydrophobic polymers can improve water solubility (Silva-Abreu et al., 2018). When PLGA is covalently attached to PEG as a carrier, the hydrophilic PEG surface coating layer with no charge has the effect of three-dimensional obstruction. The steric hindrance effect developed on the surface of NPs can block or delay the phagocytosis of drugs by the phagocytic system, and reduce the adsorption of opsin protein on the surface of NPs in the blood to achieve long circulation (Zhang et al., 2018).

### Characterization of PEG-PLGA NPs

NPs with particle sizes ranging from 1 to 1,000 nm are solid colloidal particles composed of polymer materials. It can disperse in water to form an approximately colloidal solution with high targeting (Lee et al., 2012). It has been reported that particle size is a key factor affecting the uptake of NPs by cells and is closely related to the distribution and pharmacokinetic behaviour of NPs *in vivo*. When the size of NPs is 200 nm, drugs can be delivered into the central nervous system, and NPs of about 100 nm can more easily cross the blood-brain barrier and accumulate in lesions (Costantino and Luca, 2010). It has also been reported that passive targeting of NPs in the size range of 60–400 nm is effective for tumour therapy (ONEAL, 2004). The current study showed that the size of the prepared NPs was 200 nm, which met the requirements for delivery *in vivo*. In addition, the zeta potential of PLGA-PEG-COOH NPs was negative, while the zeta potential of PLGA-PEG-COOH NPs coupled to CHP was positive. Because the long PEG chain shields part of the positive charge, the zeta potential eventually decreases. The long PEG chain could not react after coupling CHP outside the NPs, so the zeta potential was positive. The electrostatic stability measured by the zeta potential is an important parameter for characterising and monitoring the physical stability. Drug suspension is a system that disperses solid ions in a suspension medium or carrier. NPs are suspended in a liquid medium and form a colloidal suspension when dispersed in a liquid medium. When administered intravenously, changes in surface properties are an important parameter affecting the interaction and subsequent distribution and metabolism of NPs with other biological components *in vivo* (Patil, S., et al., 2007; Vogel R, et al., 2017; Smith, M. C., et al., 2017). Patil (Patil, S., et al., 2007) also found that NPs with positive zeta potential could adsorb more protein, while samples with negative zeta potential showed little or no protein adsorption. Cellular uptake studies showed the preferential uptake of negatively charged NPs. These results suggest that electrostatic interactions play an important role in the protein adsorption and cellular uptake of NPs.

### Drug Loading, Encapsulation Rate and Release of NPs

The targeting property of nanoformulations highlights a unique advantage in the treatment of cardio-cerebrovascular diseases, and carrier PEG-PLGA plays an important role in the development of innovative nanomedicines because it is safe and nontoxic. Gholaminejad A (Gholaminejad et al., 2021) found that dysregulated expression of mir-30b-5p was closely related to heart failure occurrence by bioinformatics analysis. Wang et al. (2015) discovered a novel signalling pathway composed of E2F1, miR-30b, and CypD, which could regulate myocardial necrosis and subsequently provide a new direction for the effective treatment of myocardial infarction and heart failure. Both of these studies suggest that mir-30b influences the progression of heart failure and may serve as a novel target for heart failure therapy. Due to gastrointestinal metabolism and hepatic breakdown, drugs such as miR-30b-5p reach the site of myocardial injury with reduced potency. Additionally, nucleic acid drugs are easily degraded by proteinase class, so PEG-PLGA NPs were adopted for mir-30b-5p encapsulation to reduce the degradation and destruction of drugs in non-targeted tissues.

Compared with the traditional PLGA carrier, PEG-modified PLGA changed the release rate of drugs, increased the action time of drugs in the body, and promoted the stability and targeting of NPs. Rapamycin (RAPA) has pharmacological effects in the treatment of atherosclerosis, but its water solubility is poor. RAPA-PEG-PLGA-NPs prepared using PEG-PLGA as a carrier, and the *in vitro* release assay showed that the sustained release of the NPs was as long as 5 days with a cumulative release more than 70% (Serris et al., 2020). Some researchers have prepared aspirin PEG-PLGA microspheres (Asp-PEG-PLGA-MSs), and Asp-PEG-PLGA-MSs required more than 120 h for the release of 95% of the aspirin, achieving the effect of sustained release (Lei et al., 2019; Ghavimi et al., 2020). In the current study, it was found that the mean entrapment efficiency of the mir-30b-5p NPs was 81.8 ± 2.1%, and the release of more than 90% of mir-30b-5p lasted up to 5 days The release from PEG-PLGA NPs was faster in the initial 24 h, which was mainly due to adsorption on the particle surface or the rapid release of incomplete entrapped drug. After 24 h, drug release was relatively slow. The slow release was due to the drug being embedded in the core-shell of PEG-PLGA NPs; their was entrapped in the lipophilic core and was difficult to escape, so the drug release was relatively slow.

### Safety of NPs

Although PEG-PLGA has many advantages as a carrier for nanoformulations, its safety has been a concern (Kang et al., 2014). In this experiment, the proliferation of cardiomyocytes following treatment with PEG-PLGA mir-30b-5p-loaded NPs was not found to affect cell proliferation. This result suggests that at the cellular level, PEG-PLGA is safe as a carrier for miR-30b-5p. However, its safety needs to be further explored in animals.

### Therapeutic Effects of miR-30b-5p-Loaded NPs on Heart Failure

Animal experiments showed that after tail vein injection of NPs, the fluorescence intensity was the highest in the heart and lower in the liver and kidney, indicating that the NPs were mainly distributed in the heart. PEG-PLGA NPs have a better EPR effect, which can concentrate drug-loaded NPs in the injured myocardial part, and with the help of the transmembrane transport ability of PEG-PLGA, enhance the cellular uptake of drugs to achieve precise drug delivery, and also largely reduce the administered dose (Wu et al., 2013; Pattni and Torchilin, 2015). In various animal models, such NPs released 10 times more drug at the tumour site compared to the administration of free drugs and exhibited prolonged circulation time. In addition, the effect on tumour shrinkage in animal models was much higher than that of free docetaxel (Farokhzad et al., 2006; Hrkach et al., 2012).

This study confirmed that the heart function of heart failure model rats in the miR-30b-5p-loaded NP group was significantly better than that of heart failure rats in the miR-30b-5p-non-loaded NP group. The results of HE and Masson staining also showed that the heart damage in the miR-30b-5p-loaded NP group was significantly lower than that in the miR-30b-5p-non-loaded NP group. Apoptosis assay showed that the apoptosis rate of cardiomyocytes in the heart failure model group treated with miR-30b-5p-loaded NPs was lower than that in heart failure rats treated with miR-30b-5p-non-loaded NPs. The above results indicate that miR-30b-5p-loaded PEG-PLGA NPs have a certain therapeutic effect on rats with heart failure. These results also fully illustrate the targetability of PEG-PLGA NPs and their effectiveness in heart failure treatment.

### Effects of miR-30b-5p-Loaded NPs on Myocardial Hypertrophy and Inflammation

Heart failure is a common pathological feature of many cardiovascular diseases, such as myocardial hypertrophy, acute myocardial infarction, and myocardial ischemia-reperfusion injury. Cardiac hypertrophy is an adaptive response of cardiomyocytes to increased persistent load. Initial myocardial hypertrophy has a certain compensatory significance, but cardiac hypertrophy and myocardial remodelling caused by myocardial hypertrophy can eventually lead to mental failure, so preventing cardiac hypertrophy is important for the treatment of heart failure (Wang and J., 2005; Klussmann et al., 2016). We found that compared with the model and mir-30b-5p non-loaded NPs, treatment with mir-30b-5p-loaded NPs significantly reduced the levels of cardiac hypertrophy markers (ANP, BNP, and β-MHC), suggesting that mir-30b-5p-loaded NPs have a therapeutic effect on cardiac hypertrophy. The levels of inflammatory factors IL-6 and IL-1β are positively associated with heart failure severity (Turner et al., 2007; Mao et al., 2014). Therefore, the levels of inflammatory factors can laterally reflect the severity of heart failure and also the therapeutic effect of drugs on heart failure. Compared with the model group and the miR-30b-5p-non-loaded NP group, the expression levels of inflammatory factors (IL-1β, IL-6) in the miR-30b-5p-loaded NP group decreased significantly.

### Molecular Mechanism of miR-30b-5p-Loaded in NPs in Heart Failure

Multiple studies have addressed the role of miR-30b-5p in tumours and the associated mechanisms of action. Liu et al. (2017) identified miR-30b-5p as a novel tumour suppressor that regulates renal cell carcinoma cell proliferation, metastasis, and epithelial mesenchymal transition (EMT) by downregulating GNA13 expression. In addition, therapeutic effects have also been reported in gliomas (Qin et al., 2017), inflammatory diseases (Yang et al., 2020), liver cancer (Qin et al., 2017; Zhang et al., 2020), and other diseases. We found that there was a binding site of miR-30b-5p with TGFBR2 using bioinformatics software as well as dual luciferase activity assay experiments.

TGF-β is a member of the TGF superfamily and plays an important role in maintaining tissue homeostasis and repair, as well as in certain tumours, vascular diseases, and fibrosis diseases (Lim and Zhu, 2006; Benjamin, 2012; Ni et al., 2017). TGF-β and related signalling pathways also play an important role in the process of heart failure (Song and Wang, 2015; Chen et al., 2018).TGFBR2 is a type II receptor of TGF-β, which contains an intracellular serine/threonine kinase domain. TGF-β must bind to specific receptors on the cell membrane, including TGFBR2, to play a biological role. Our results showed that NPs loaded with miR-30b-5p could regulate the expression of certain inflammatory factors by lowering TGFBR2, and then achieve the purpose of treating heart failure.

## Conclusion

In conclusion, PEG-PLGA NPs loaded with miR-30b-5p can inhibit apoptosis, fibrosis, and myocardial injury, regulate the expression of related inflammatory factors, and then treat heart failure by targeting PEG-PLGA. Although PEG-PLGA has demonstrated significant efficacy in drug delivery systems, it is limited to the animal experimental stage, and clinical trials are needed to determine its pharmacokinetics and pharmacodynamics.

## Data Availability

The original contributions presented in the study are included in the article/Supplementary Material, further inquiries can be directed to the corresponding author.
